# Content-illumination coupling guided low-light image enhancement network

**DOI:** 10.1038/s41598-024-58965-0

**Published:** 2024-04-11

**Authors:** Ruini Zhao, Meilin Xie, Xubin Feng, Xiuqin Su, Huiming Zhang, Wei Yang

**Affiliations:** 1 Key Laboratory of Space Precision Measurement Technology, Xi’an Institute of Optics and Precision Mechanics, Chinese Academy of Sciences, Xian, 710119 China; 2 Institute of Intelligent Transportation, Shandong Provincial Communications Planning and Design Inst Group Co., Ltd., Jinan, 250101 China; 3 Chang’an University, Xian, 710064 China; 4 Pilot National Laboratory for Marine Science and Technology, Qingdao, 266200 China

**Keywords:** Low-light enhancement, Retinex, End-to-end, Truss topology, Pre- and post-activation, Mathematics and computing, Optics and photonics

## Abstract

Current low-light enhancement algorithms fail to suppress noise when enhancing brightness, and may introduces structural distortion and color distortion caused by halos or artifacts. This paper proposes a content-illumination coupling guided low-light image enhancement network (CICGNet), it develops a truss topology based on Retinex as backbone to decompose low-light image component in an end-to-end way. The preservation of content features and the enhancement of illumination features are carried out along with depth and width direction of the truss topology. Each submodule uses the same resolution input and output to avoid the introduction of noise. Illumination component prevents misestimation of global and local illumination by using pre- and post-activation features at different depth levels, this way could avoid possible halos and artifacts. The network progressively enhances the illumination component and maintains the content component stage-by-stage. The proposed algorithm demonstrates better performance compared with advanced attention-based low-light enhancement algorithms and state-of-the-art image restoration algorithms. We also perform extensive ablation studies and demonstrate the impact of low-light enhancement algorithm on the downstream task of computer vision. Code is available at: https://github.com/Ruini94/CICGNet.

## Introduction

The low-light enhancement algorithm has very broad application prospects in fields such as intelligent driving and intelligent security. For the environmental perception technology involved, most of them are based on sufficient illumination. Most perception algorithms are not suitable for the case of insufficient illumination. To improve the safety of intelligent driving and the accuracy of intelligent security, the basic goal is to restore the degraded scene to be recognized.

Most of the early methods are based on histogram equalization to enhance the brightness and contrast of low-light images^1,2^. Histogram equalization will cause grayscale overlap, loss of local details, obvious block effects when merging gray levels. This type of methods is a global enhancement method, which cannot effectively improve local contrast, and shows poor enhancement effect on images with uneven illumination. The local equalization performed on different spatial regions^3^ usually has a greater impact on the average brightness. Subsequently, Retinex theory was proposed to decompose the reflectance component and illumination component of images, and subsequent low-light enhancement algorithms improve the classic histogram equalization and Retinex-based methods in many ways. Due to the uncertainty of the initial position, end position and path selection, the path-based Retinex algorithms^4^ are easy to introduce unnecessary noise. They also show higher computational complexity, they are difficult to prevent color distortion. The center/surround-based Retinex algorithms^5^ need to set multiple uncertainty parameters, resulting in uncertainty in the contrast, chroma, sharpness of the enhanced image. The original Retinex-based algorithms and the subsequent improved algorithms involving color distortion need to obtain the illumination map. Most of the priors used to estimate the illumination map are artificially set, these methods show poor generalization. Most Retinex-based learning methods^6^ use a two-stage strategy to achieve low-light enhancement, Retinex is generally used for pre-processing in the first-stage. Learning-based low-light enhancement algorithms fail to suppress noise, and cannot eliminate noise or even enhance noise when enhancing illumination. Meanwhile, the current algorithms cannot estimate global and local illumination simultaneously, inaccurate illumination estimation will cause halos and artifacts, which will cause color or structural distortion.

**Figure 1:**
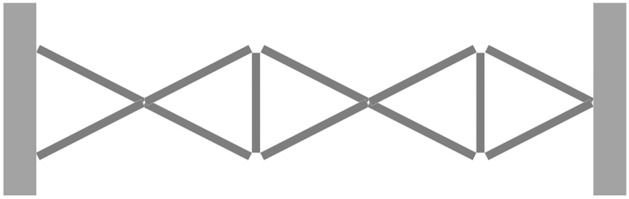
Schematic diagram of truss topology.

The main contributions of proposed network are as follows:

- Inspired by the use of pre-activation features as optimization item in super-resolution tasks, it is expected to provide stronger supervision for the network, our proposed network develops a cascaded multi-residual architecture (CMRA) using pre- and post-activation features at different depth levels, it improves the reusability of features.
- Proposed network uses a truss topology as backbone, it is shown as Fig. 1, which is integrated into Retinex in an end-to-end way. Proposed network performs multiple decompositions of content-illumination feature and reconstruction of enhanced features along with depth and width directions of truss topology.
- This paper explores the effects of low-light enhancement algorithms on semantic segmentation performance under different data distributions and data amounts, that’s, low-level image reconstruction tasks serve high-level visual perception tasks under different application conditions.

## Related work

### Traditional retinex-based methods

Yue et al.^7^ combine both reflectance and illumination layers to perform image decomposition, they regularize the illumination layer so that the decomposed reflectance would not be affected much by illumination. Fu et al.^8^ propose a weighted variational model to estimate both the reflectance and the illumination, the model could preserve the estimated reflectance with more details. Zhang et al.^9^ consider exposure correction problems as an illumination estimation optimization, they also leverage perceptually bidirectional similarity to generate the desired result with even exposure, vivid color and clear textures. Cai et al.^10^ propose a joint intrinsic-extrinsic prior model to estimate both illumination and reflectance, the model could preserve the structure information by shape prior, estimate reflectance with texture prior and capture illumination information based on illumination prior. Gao et al.^11^ propose a naturalness preserved illumination estimation algorithm by a joint edge-preserving filter. The proposed algorithm could comprehensively take all the constraints into consideration, including spatial smoothness, sharp edges on illumination boundaries. Li et al.^12^ propose a robust Retinex model considering a noise map to improve the performance of enhancing low-light images with intensive noise.

### Retinex-based learning methods

Zhang et al.^13^ decompose images into two components, one component is used for illumination adjustment, the other is used for degradation removal. Zhao et al.^14^ propose a generative strategy for Retinex decomposition, they also propose a network to estimate latent component for low-light enhancement, proposed method could reduce the coupling relationship between illumination and reflectance component. Liu et al.^15^ construct a model to represent the intrinsic underexposed structure of low-light images, they also design a cooperative reference-free learning strategy to search low-light prior architecture from a compact search space. Lu et al.^16^ propose a two-branch exposure-fusion network to deal with blind low-light enhancement, they leverage an enhancement strategy to estimate the transfer function for varied illumination levels. They also introduce a generation-and-fusion strategy to enhance slightly and heavily distorted images. Zhu et al.^17^ propose a three-branch network to deal with illumination, reflectance and noise based on Retinex respectively, they also design a zero-shot scheme to iteratively minimize loss function. Hui et al.^18^ propose a decomposition network to decompose the image into reflectance and illumination maps, they enhance two maps separately. They also propose an adaptive residual feature block to leverage the feature correlation between low-light and normal-light images. Hui et al.^19^ leverage a detail component prediction model to obtain detail enhancement component, they propose a decomposition network to decompose V-channel into reflectance map and illumination map, the enhancement component is used to enhance the reflectance map.

### Other learning methods

Jin et al.^20^ propose an event-guided low light enhancement network, the generator contains image enhancement branch for enhancing low-light image and a gradient reconstruction branch for learning gradient from events. Cai et al.^21^ propose a network with a higher compression rate and better enhancement performance for low-light images, the network is a two-branch architecture with lower computational cost, one is main enhancement branch, the other is signal-to-noise aware branch. MBPNet^22^ consists of four different branches which map the relationship at different scales, the network leverages a progressive enhancement strategy, it also embeds long short-term memory networks in four branches for iteratively performing the enhancement process. Han et al.^23^ propose a dual-branch fusion low-light image enhancement, the upper branch is a refinement branch focusing on noise suppression, and the lower branch is a U-Net-like global reconstruction branch for high-quality image generation. Lv et al.^24^ propose a low-light enhancement network with four branches, in which Attention-Net is used to estimate the illumination to guide the method to pay more attention to the underexposed areas, Noise-Net is used to guide the denoising process, Enhancement-Net can simultaneously enhance and denoise, the Reinforce-Net is used for contrast re-enhancement. Lu et al.^25^ propose a multi-branch topology residual block-based network, the network increases the width of the network and enhances information delivery along with the depth and width directions.

Current low-light enhancement algorithms fail to suppress noise when enhancing brightness, and may also introduces structural distortion and color distortion caused by halos or artifacts. Our proposed low-light enhancement network is expected to enhance the illumination component and maintain the content illumination by stage-by-stage learning. Each submodule uses the same resolution input and output to avoid the introduction of noise. The illumination component in the initial stage focuses on global illumination features, subsequent stages pay more attention to local features to prevent color distortion caused by the halo and inaccurate illumination estimation. We use a multi-space pyramid content learning module to adaptively adjust the content features based on stage-by-stage illumination components to prevent structural distortion.

## Methodology

**Figure 2:**
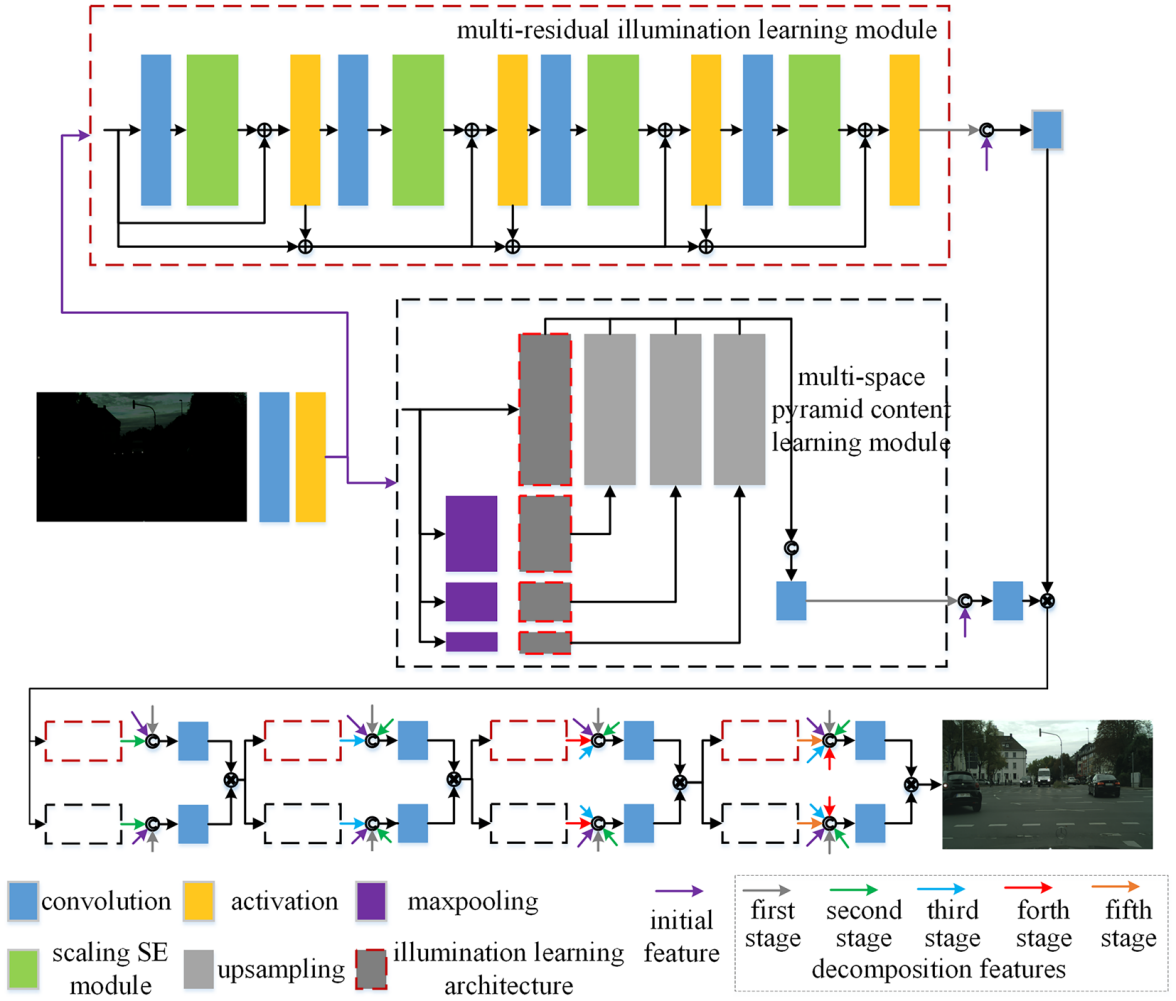
Overall architecture of CICGNet.

We propose a content-illumination coupling guided low-light image enhancement network (CICGNet), it is shown as Fig. 2, CICGNet develops a truss topology as backbone and integrates Retinex in an end-to-end way. The proposed network decomposes low-light samples and reconstructs normal light samples based on Retinex. Retinex decomposes the any input sample into illumination component and reflectance component. The reflectance component is the color of the object itself and has nothing to do with the intensity or illumination. We regard the reflectance component as the content component of the sample. CICGNet regards the low-light enhancement task as the enhancement of illumination component and the maintenance of the content component.

The initial features of the low-light samples are decomposed along with deep and width directions of truss topology. The feature decomposition and reconstruction of the network are iterated for many times based on the truss topology. All extracted features of the previous stage are integrated into the subsequent stage. Meanwhile, the multi-residual illumination learning module is used to enhance the reusability of pre- and post-activation features, multi-space pyramid content learning module is used to enhance the reusability of pre- and post-activation features and multi-level features at different depth levels.

After the shallow features of low-light images are extracted and activated, they are sent to the multi-residual illumination learning module and multi-space pyramid content learning module along with truss rod, respectively. The shallow features are extracted using 3 × 3 convolution kernel, stride is 1, padding is 1, the output channel is 32, ReLU is used for nonlinear activation. Above two modules will be introduced in detail below.

### Cascaded multi-residual illumination learning module

**Figure 3:**
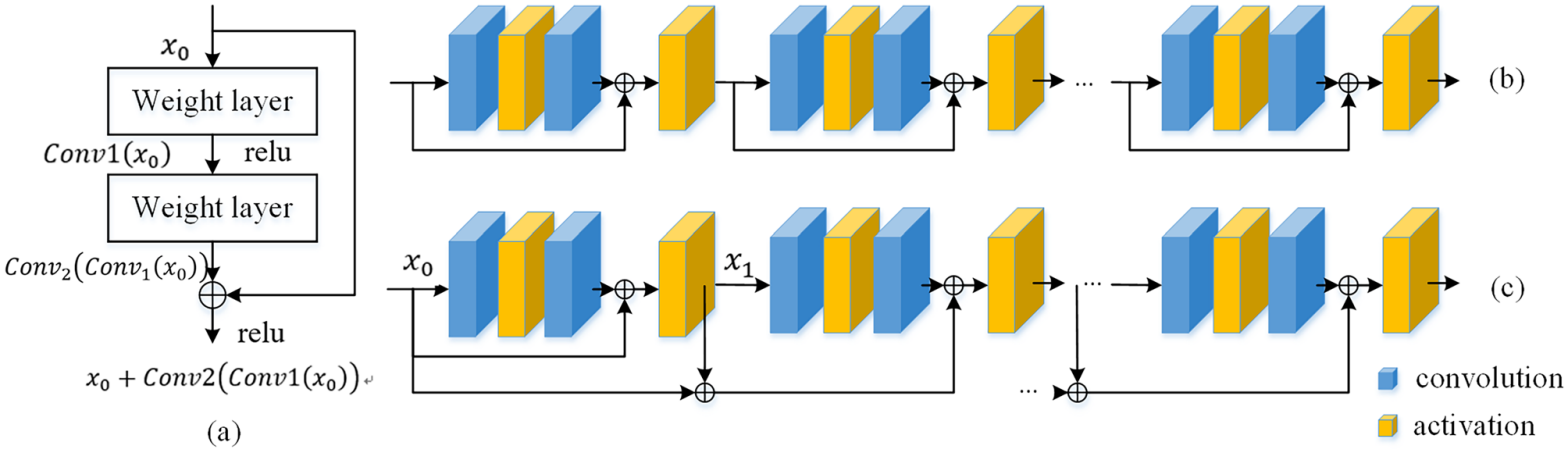
(**a**) Classic residual architecture. (**b**) classic cascaded residual architecture (CRA). (**c**) proposed CMRA.

Layers at different depth can extract feature under different receptive fields, extracted feature show different roles in different tasks. As the depth of the network increases, gradient is prone to disappear when passing through multiple layers of backpropagation. Meanwhile, the increase in model depth will cause network performance to decrease rather than increase. To solve this problem, deep residual network^26^ establishes a direct mapping between low-level features and high-level features through skip connections. Classic residual architecture is shown as Fig. 3a, the input ${x}_{0}$ is directly applied to the output ${Conv}_{2}\left({Conv}_{1}\left({x}_{0}\right)\right)$ through skip connection. It enables deep layers to take advantages of extracted features from shallow layer, makes the information transmission more complete and increases the reusability of information. It can be used to improve gradient disappearance and significantly improve network performance. Ignoring the activation function, residual blocks are shown in Eq. (1). The two convolution operations in residual blocks are shown in Eq. (2).

1 $$\begin{array}{c}{x}_{1}={Conv}_{1}\left({x}_{0}\right)+{x}_{0},\end{array}$$

2 $$\begin{array}{c}{x}_{1}={Conv}_{2}\left(({Conv}_{1}\left({x}_{0}\right)\right)+{x}_{0},\end{array}$$

Multiple residual blocks are used for cascaded feature extraction, as shown in Fig. 3b, this section improves the cascaded residual blocks on this basis. As shown in Eq. (3) and (4), the original residual network directly maps the input ${x}_{0}$ in the output of a residual block ${ResBlock}_{1}$. As shown in Eq. (7), the input to the nth residual block ${ResBlock}_{n}$ is ${x}_{n-1}$. Similarly, as shown in Eq. (8), the output of the previous residual block ${x}_{n-1}$ is mapped to ${x}_{n-0}$ before nonlinear fitting is performed.

3 $$\begin{array}{c}{x}_{1\_0}={ResBlock}_{1}\left({x}_{0}\right),\end{array}$$

4 $$\begin{array}{c}{x}_{1}=ReLU\left({x}_{1\_0}+{x}_{0}\right),\end{array}$$

5 $$\begin{array}{c}{x}_{2\_0}={ResBlock}_{2}\left({x}_{1}\right),\end{array}$$

6 $$\begin{array}{c}{x}_{2}=ReLU\left({x}_{2\_0}+{x}_{1}\right),\end{array}$$

7 $$\begin{array}{c}{x}_{n\_0}={ResBlock}_{n}\left({x}_{n-1}\right),\end{array}$$

8 $$\begin{array}{c}{x}_{n}=ReLU\left({x}_{n\_0}+{x}_{n-1}\right),\end{array}$$

As the depth of the network increases, there are more combinations of features at different levels. To further improve the feature expression ability of residual architecture, this section improves on the classic cascaded residual architecture and proposes CMRA. Inspired by the use of pre-activation features as a loss function in super-resolution task to optimize the network. This loss function takes into account that the activated features are very sparse as the depth of the network increases. For the classic baboon image in super-resolution task, the activated neurons only account for 11.17% with VGG19-54^27^. Considering that the sparse features are not enough to provide strong supervision for the network. For the proposed multi-residual architecture, in addition to using the post-activation features of the previous residual module, combined with the pre-activation features of the previous residual module, a multi-residual mapping module is formed. As shown in Fig. 3c, taking the nth multi-residual architecture (MRA) as an example, in addition to integrating the input ${x}_{n\_0}$ of the current stage and the activated output ${x}_{n-1}$ of previous stage, the MRA needs to combine the input ${x}_{n-2}$ before activation of the previous stage, as shown in Eq. (10). Instead of using a full residual connection that would cause the model to be too large, the proposed cascaded multi-residual architecture can reduce the computational complexity of the model, and obtain multiple sets of pre-activation and post-activation features at different depth levels.

9 $$\begin{array}{c}{x}_{2}=ReLU\left({x}_{2\_0}+{x}_{1}+{x}_{0}\right),\end{array}$$

10 $$\begin{array}{c}{x}_{n}=ReLU\left({x}_{n\_0}+{x}_{n-1}+{x}_{n-2}\right).\end{array}$$

**Figure 4:**
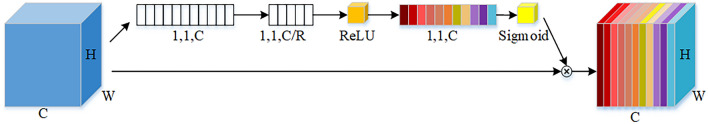
Scaling SE module.

As shown in Fig. 2, the red dashed box is a multi-residual illumination learning module, which is used to extract the illumination component. The input and output channels of the blue convolutional block in this module are both 32, the kernel size is 3 × 3, stride is 1, padding is 1. For the specific parameters in the scaling Squeeze-Excitation (SE) module, as shown in Fig. 4, the spatial features are compressed using adaptive averaging pooling, the channel scaling factor $R$ is 4, and the channel features are fitted nonlinearly using the ReLU. For the nonlinear fitting at the end of each residual module, we use LeakyReLU to preserve the neuronal activation values of the positive and negative regions. The Sigmoid is used to map the output of the module into probability to weight the initial features.

### Multi-space pyramid content learning module

**Figure 5:**
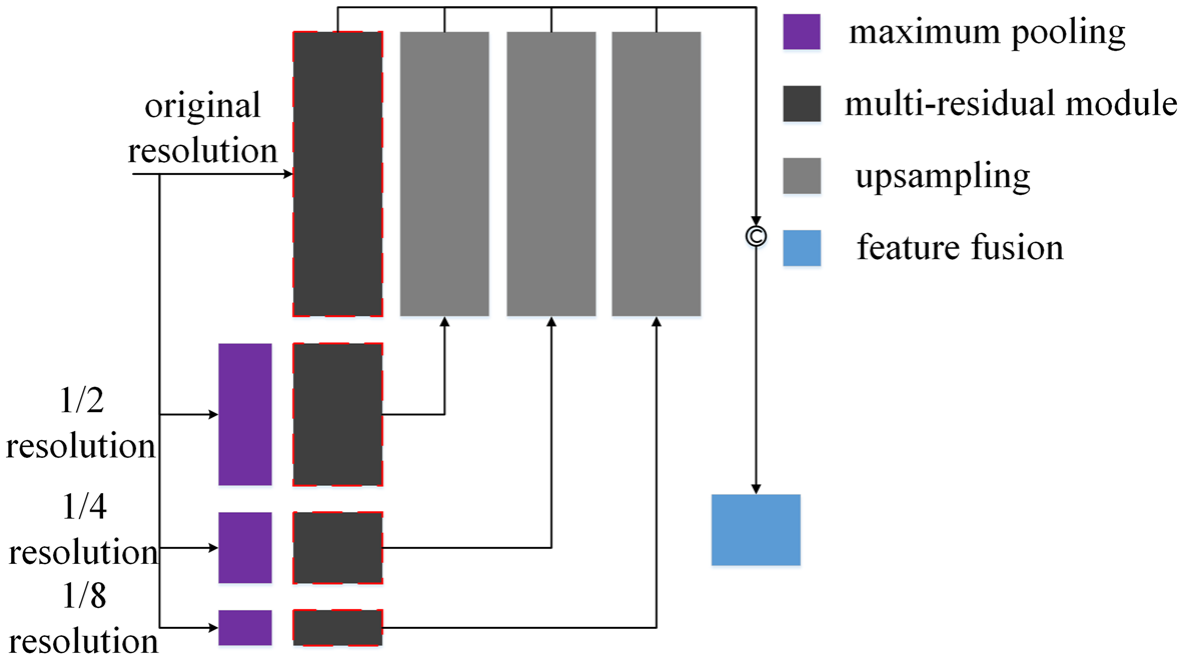
Multi-space pyramid content learning module.

Aiming at the maintenance of content features, as shown in Fig. 5, we propose a multi-space pyramid content learning module. Inspired by the good performance of pyramid architecture on various computer vision tasks, to capture different content details, we use pyramid structure to obtain the features of the same instance at different resolutions. Specifically, we use maximum pooling to obtain features of 1/2, 1/4 and 1/8 resolution, respectively. The CMRA proposed in the illumination learning module is used to enhance features of different scales, that is, the architecture consistent with the illumination learning module is used for the four spaces of the feature pyramid. As shown in Fig. 5, the gray block with red dashed lines in content learning module uses the same architecture as the illumination learning module. While enhancing the reusability of pre- and post-activation features at different depth levels, it is also used to enhance the reusability of multi-space features. The construction and enhancement of multi-space features are shown in Eqs. (11)–(16).

11 $$\begin{array}{c}{F}_{0}=CMRA\left(F\right),\end{array}$$

12 $$\begin{array}{c}{F}_{1/2}=CMRA\left(MaxPool\left(F,\left(H/2\right),\left(W/2\right)\right)\right),\end{array}$$

13 $$\begin{array}{c}{F}_{1/4}=CMRA\left(MaxPool\left(F,\left(H/4\right),\left(W/4\right)\right)\right),\end{array}$$

14 $$\begin{array}{c}{F}_{1/8}=CMRA\left(MaxPool\left(F,\left(H/8\right),\left(W/8\right)\right)\right),\end{array}$$

where $CMRA$ represents cascaded multi-residual architecture, $MaxPool$ is maximum pooling, $H$ and $W$ represent height and width of initial features. After enhancing the features at the four spaces respectively, bilinear interpolation is used to restore the feature resolution. Then we use dense connections to splice features at four scales according to channel. For spliced features, multiple convolution kernels are used to extract the features under the extended channel. For the spliced multi-scale content features, as shown in Eq. (16), we use channel compression strategy to model the complementary or redundant relationship of the multiple channels, this way can obtain the output of final content learning module.

15 $$\begin{array}{c}F=Concat,\end{array}$$

16 $$\begin{array}{c}{out=Conv}_{1\times 1}\left(F\right),\end{array}$$

where $Up$ represents bilinear interpolation, $Concat$ indicates splicing by channel.

### Feature decomposition and reconstruction

**Figure 6:**
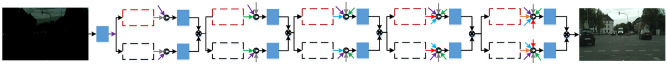
Multi-stage feature decomposition and reconstruction architecture.

As shown in Fig. 6, the proposed CICGNet contains several times of feature decomposition and reconstruction along with truss topology. As mentioned above, the initial features of low-light images are sent into the illumination learning module and content learning module to enhance illumination feature and maintain content feature respectively. The red and black dashed boxes in Fig. 6 represent illumination learning module and content learning module. Each stage of feature decomposition and reconstruction will incorporate the features of previous stage to form an adaptive multi-feature fusion. The initial features, decomposition features of the first, second, third, fourth, fifth stages of the low-light image are represented by purple, gray, green, blue, red and orange lines respectively. In the five-time feature decomposition and reconstruction based on Retinex, the network always maintains the content feature component and gradually enhances the illumination feature components, it finally obtains an enhanced image that meets the visual effect.

### Loss function

To realize low-light enhancement task, we consider structural distortion, content loss and uneven illumination condition, we combine structural loss (${L}_{str}$), content loss (${L}_{con}$) and illumination region loss (${L}_{reg}$) to optimize the proposed CICGNet as shown in Eq. (17). We use structure similarity index measure (SSIM) and multi-scale SSIM (MS-SSIM) to constrain structural distortion, it is shown as Eq. (18). We leverage trained VGG19 on ImageNet to extract content feature of enhanced image and ground truth, then we use L1 loss to constrain extracted feature to prevent content loss, it is shown as Eq. (19). We use the illumination region loss^28^ to deal with uneven illumination, it is shown as Eq. (20)

17 $$\begin{array}{c}L={L}_{str}+{L}_{con}+{L}_{reg},\end{array}$$

18 $$\begin{array}{c}{L}_{str}={2-L}_{ssim}-{L}_{ms-ssim},\end{array}$$

19 $$\begin{array}{c}{L}_{content}={\Vert VGG(G\left({x}_{ij}\right))-VGG(GT))\Vert }_{1},\end{array}$$

20 $$\begin{array}{c}{L}_{region}=4\cdot \frac{1}{w\cdot h}\sum_{i=1}^{w}\sum_{j=1}^{h}\left({\Vert {G}_{L}\left({x}_{ij}\right),{GT}_{L}\Vert }_{1}\right)+\frac{1}{w\cdot h}\sum_{i=1}^{w}\sum_{j=1}^{h}\left({\Vert {G}_{H}\left({x}_{ij}\right),{GT}_{H}\Vert }_{1}\right),\end{array}$$

where $w$ and $h$ represent width and height of input low-light image, ${G}_{L}\left({x}_{ij}\right)$ and ${GT}_{L}$ are low-light part of enhanced image and its corresponding ground truth, ${G}_{H}\left({x}_{ij}\right)$ and ${GT}_{H}$ are rest part of enhanced image and its corresponding ground truth.

## Experiments and results

### Datasets and experimental details

We choose three real low-light enhancement datasets (LOL^29^, LSRW^30^ and VE-LOL-L^31^) and two synthetic low-light enhancement datasets (BrighteningTrain^32^ and CityscapesL^33^) to evaluate our proposed CICGNet. LOL is the first truly captured paired low-light enhancement dataset, collected by varying exposure time and ISO, and image registration is applied to the captured images. The dataset contains 485 training pairs and 15 test pairs. LSRW is captured using Nikon D7500 and HUAWEI P40 Pro, again by varying exposure time and ISO to obtain pairs of images. The ISO for low light condition is 50 and ISO for normal light condition is fixed at 100. The dataset contains a total of 5600 training pairs and 50 testing pairs. VE-LOL-L is a subset of VE-LOL applied to low-level visual tasks. We use 400 pairs and 100 pairs as training samples and test samples in VE-LOL-L-Cap-Full. BrighteningTrain performs low-light synthesis on the Raw images of RAISE, the synthesis process takes into account the degradation process of low-light images and combines the statistical characteristics of natural images. It contains 900 pairs and 100 pairs as training samples and test samples.

We compare our proposed CICGNet with six state-of-the-art low-light enhancement algorithms, including HDRNet^34^, three attention-based methods ALEN^35^, SARN^36^ and ABSGNet^37^, and two latest advanced low-level image translation methods, MPRNet^38^ and Restormer^39^. As mentioned above, all comparative experiments are performed on three real datasets and two synthetic datasets. For fair comparison, all methods are retrained on five datasets.

We perform all experiments on Tesla A100. We use AdamW as optimizer, the learning rate is adjusted using cosine annealing decay. The initial learning rate is 5 × 10^–4^, the minimum learning rate decays to 5 × 10^–6^, batch size is 4. For all experiments, the training samples are randomly cropped into 256 × 256 patches and horizontally flipped with a probability of 0.5. Due to Restormer’s high computational complexity, its training samples are randomly cropped into 200 × 200, it also does not use the progressive learning strategy.

### Quantitative evaluation

In this section, we report quantitative evaluation results on five low-light enhancement datasets, including three real low-light enhancement datasets and two synthetic low-light enhancement datasets. We choose peak signal to noise ratio (PSNR), SSIM, learned perceptual image patch similarity (LPIPS)^40^, color difference metric deltaE^41^ and universal quality image index (UQI)^42^ as evaluation metrics. We give quantitative results on five low-light enhancement datasets from Tables 1, 2, 3, 4 and 5. All tables give average values for corresponding test datasets. The upward arrow represents that the higher the value, the better the network performance.

**Table 1:** The quantitative evaluation of LOL (15 images).

Methods\index	PSNR↑	SSIM↑	LPIPS↓	deltaE↓	UQI↑
HDRNet	18.095	0.794	0.189	14.463	0.872
ALEN	17.514	0.791	0.344	14.972	0.856
SARN	20.573	0.864	**0.073**	11.803	0.900
ABSGNet	20.437	0.858	0.125	11.310	0.895
**CICGNet**	*22.420*	**0.894**	**0.073**	8.940	0.921
MPRNet	22.388	*0.887*	0.087	**8.596**	**0.925**
Restormer	**22.920**	0.884	*0.076*	*8.747*	*0.922*

Bold represents the optimal value, italics indicates the sub-optimal value.

**Table 2:** The quantitative evaluation of LSRW (50 images).

Methods\index	PSNR↑	SSIM↑	LPIPS↓	deltaE↓	UQI↑
HDRNet	18.685	0.669	*0.144*	13.655	0.889
ALEN	19.603	**0.720**	0.215	12.753	*0.896*
SARN	18.960	0.685	**0.122**	13.419	*0.896*
ABSGNet	19.085	0.716	0.202	13.249	0.889
**CICGNet**	19.630	0.716	0.163	**12.242**	**0.898**
MPRNet	*19.677*	*0.719*	0.223	12.564	0.893
Restormer	**19.739**	0.718	0.180	*12.506*	0.893

Bold represents the optimal value, italics indicates the sub-optimal value.

**Table 3:** The quantitative evaluation of VE-LOL (100 images).

Methods\index	PSNR↑	SSIM↑	LPIPS↓	deltaE↓	UQI↑
HDRNet	18.466	0.827	0.196	16.311	0.886
ALEN	19.180	0.755	0.537	13.822	0.894
SARN	20.641	0.835	0.138	12.070	0.926
ABSGNet	19.190	0.818	0.280	14.920	0.895
**CICGNet**	*21.778*	**0.903**	**0.070**	*11.067*	*0.934*
MPRNet	20.438	*0.872*	0.134	13.177	0.918
Restormer	**22.185**	0.866	*0.115*	**10.986**	**0.936**

Bold represents the optimal value, italics indicates the sub-optimal value.

**Table 4:** The quantitative evaluation of BrighteningTrain (100 images).

Methods\index	PSNR↑	SSIM↑	LPIPS↓	deltaE↓	UQI↑
HDRNet	20.402	0.882	0.121	11.810	0.896
ALEN	21.453	0.875	0.153	10.331	0.920
SARN	24.072	0.940	0.038	7.988	0.939
ABSGNet	22.989	0.929	0.052	9.198	0.929
**CICGNet**	*25.530*	**0.956**	**0.027**	*7.080*	*0.951*
MPRNet	24.468	0.943	0.040	7.953	0.939
Restormer	**25.833**	*0.953*	*0.031*	**6.586**	**0.953**

Bold represents the optimal value, italics indicates the sub-optimal value.

**Table 5:** The quantitative evaluation of CityscapesL (500 images).

Methods\index	PSNR↑	SSIM↑	LPIPS↓	deltaE↓	UQI↑
HDRNet	21.118	0.822	0.274	14.829	0.896
ALEN	24.361	0.889	0.176	11.089	0.924
SARN	23.104	0.870	0.183	13.135	0.908
ABSGNet	24.729	0.896	0.168	10.712	0.927
**CICGNet**	25.383	*0.901*	**0.162**	9.816	*0.936*
MPRNet	*25.495*	0.889	*0.165*	*9.606*	0.928
Restormer	**26.169**	**0.905**	**0.162**	**8.835**	**0.944**

Bold represents the optimal value, italics indicates the sub-optimal value.

PSNR measures the quality of signal reconstruction through the mean square error. The larger the PSNR, the less distortion between two samples. SSIM is more in line with the intuitive feeling of the human eye, it mainly considers brightness, contrast and structure. The larger the SSIM, the higher the similarity between two samples. LPIPS serves as a perceptual model, it learns to generate a reverse mapping between sample and its ground truth. The lower the LPIPS, the more similar the two samples are. DeltaE is used to measure the color retention under image restoration tasks. The smaller the deltaE, the smaller the color difference. UQI mainly measures image differences based on correlation loss, contrast loss and brightness distortion. UQI is highly consistent with subjective quality indicators. The larger the UQI, the more similar the two images are.

On the premise of ensuring low-light enhancement performance, we give comparison of computational complexity, CPU/GPU inference time and network performance in Table 6. We present a comparison of MPRNet, Restormer, and CICGNet, which perform better on five low-light enhancement datasets. The computational complexity and inference time are calculated on 256 × 256. The calculation of computational complexity uses ptflops package. The running environments of CPU and GPU inference time are Intel i7-8750H CPU with 16 GB RAM and Tesla A100 respectively. As shown in Table 6, our proposed CICGNet not only achieves the optimal SSIM on these three datasets, but also shows obvious advantages in CPU and GPU inference time and computational complexity.

**Table 6:** Comparison of computational complexity, inference time and SSIM.

Methods\index	SSIM↑			CPU inference time↓/second	FLOPs↓/G	GPU inference time↓/second
	LOL	VE-LOL	BrighteningTrain			
MPRNet	0.887	0.872	0.943	2.692	148.55	0.034
Restormer	0.884	0.866	0.953	8.776	140.99	0.252
**CICGNet**	**0.894**	**0.903**	**0.956**	**1.622**	**32.65**	**0.032**

Bold indicates the optimal value.

### Qualitative evaluation

**Figure 7:**
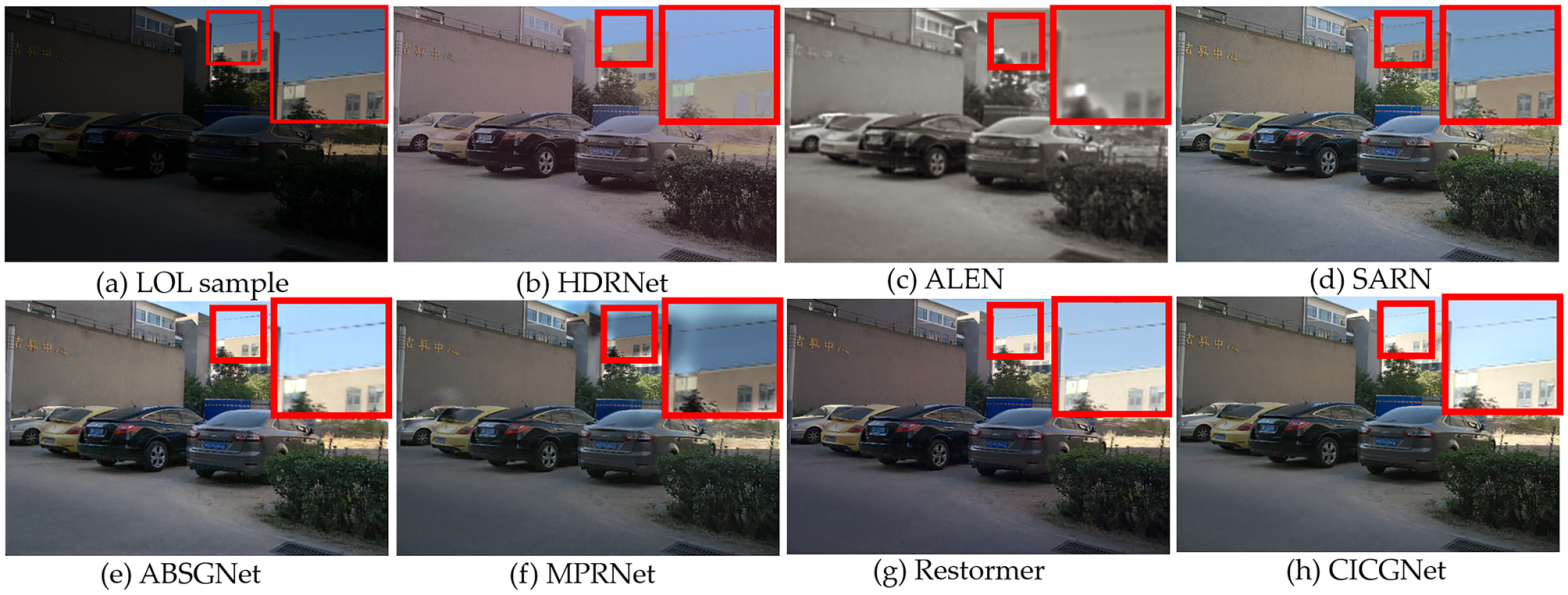
Visual results of low-light enhancement on LOL.

**Figure 8:**
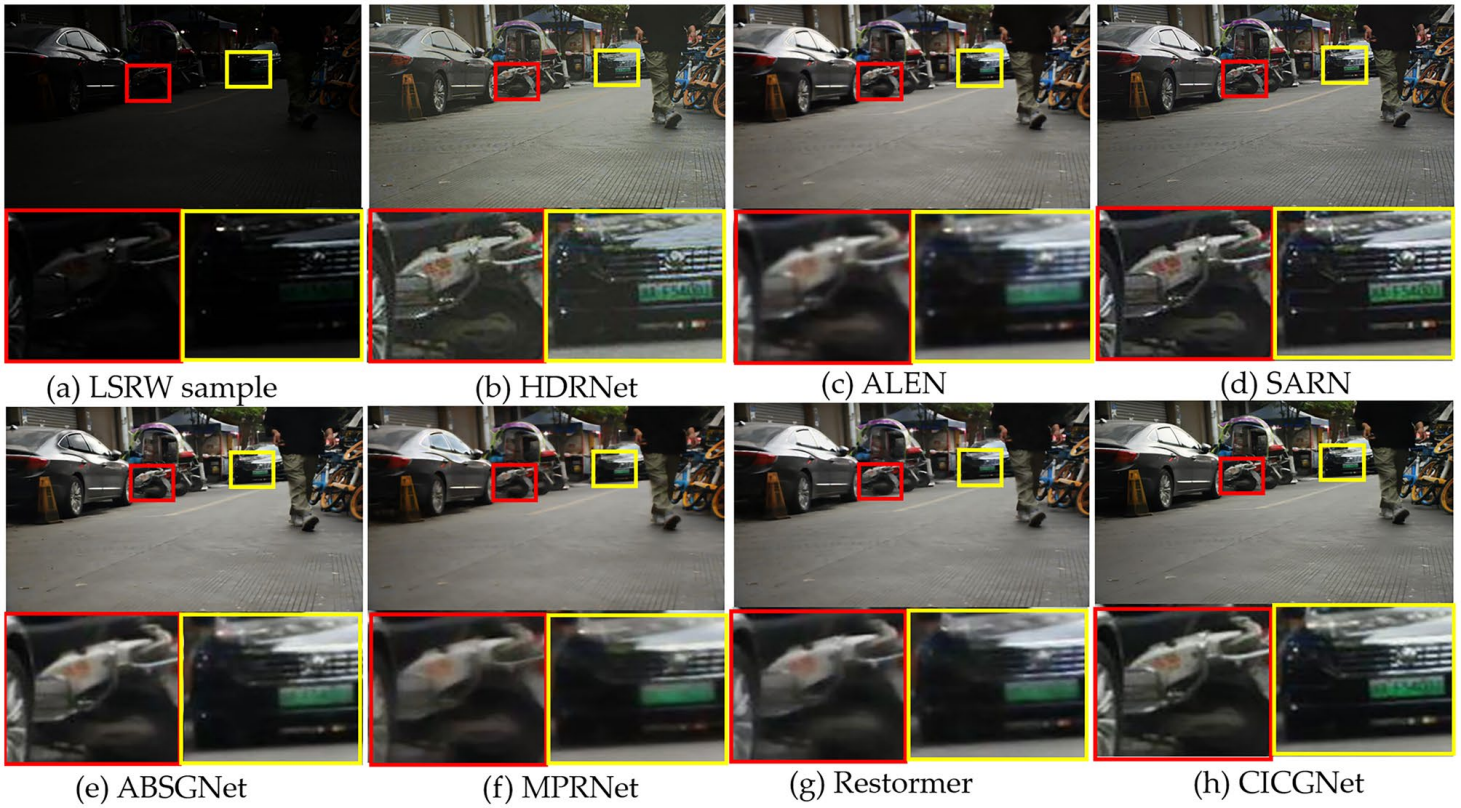
Visual results of low-light enhancement on LSRW.

**Figure 9:**
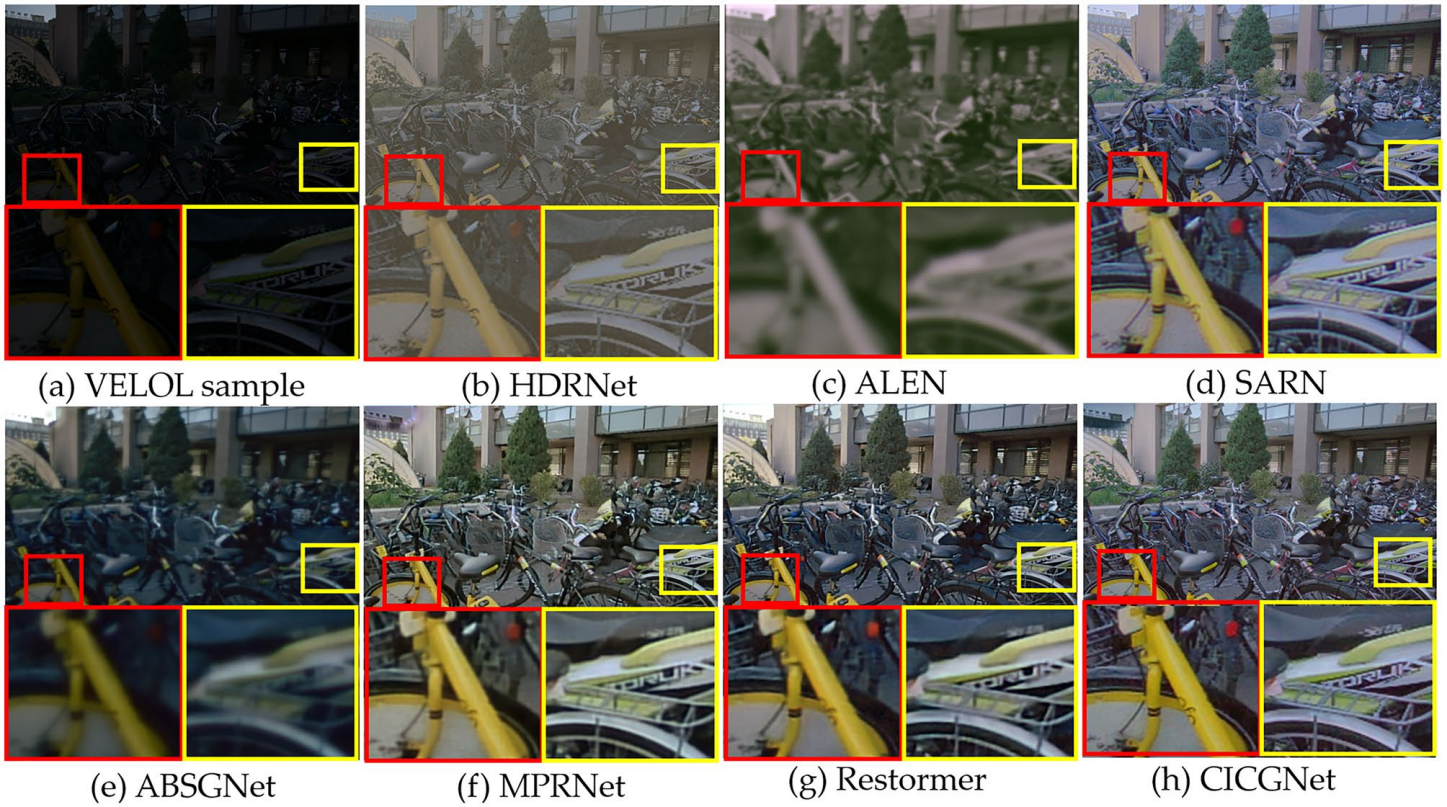
Visual results of low-light enhancement on VELOL.

**Figure 10:**
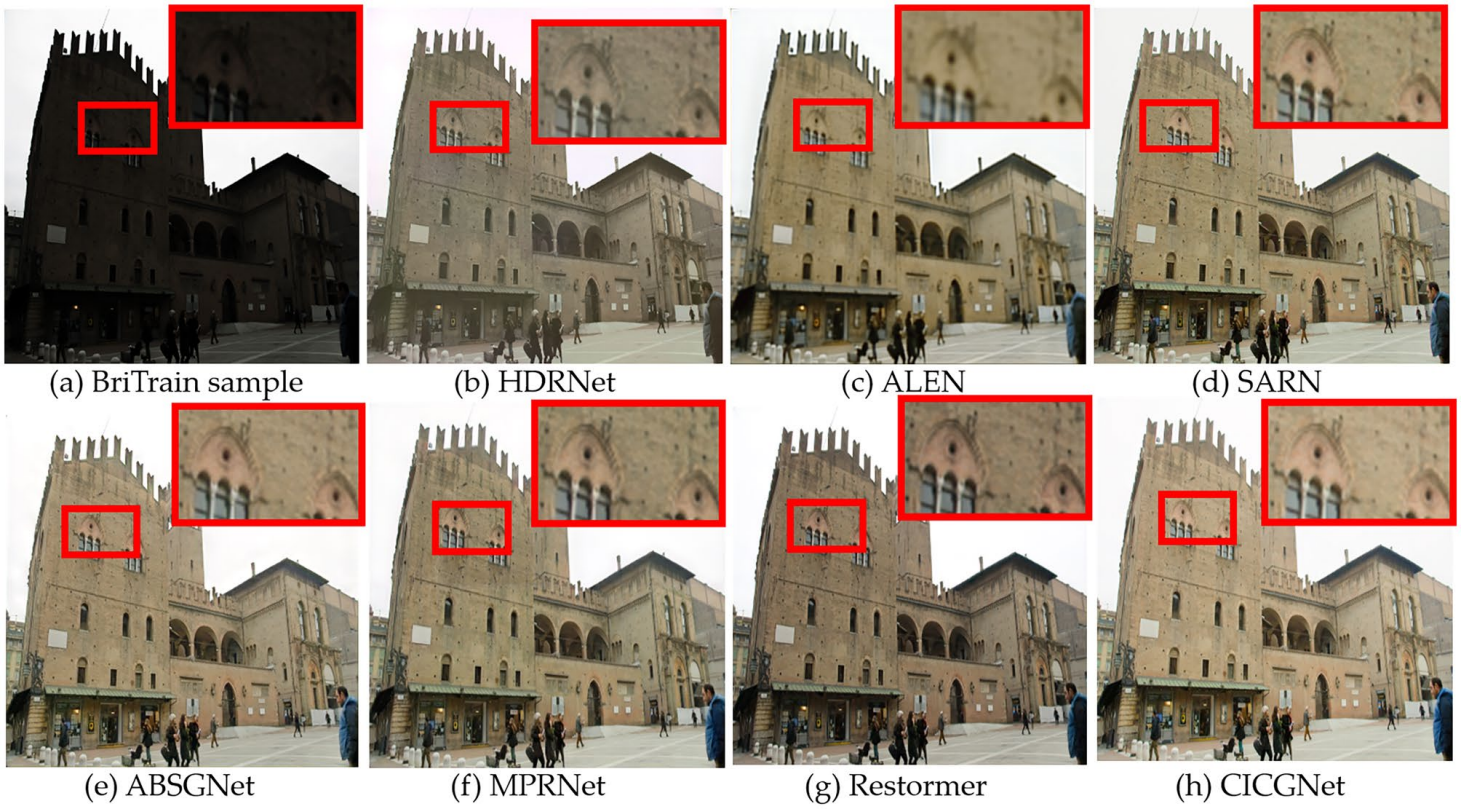
Visual results of low-light enhancement on BrighteningTrain.

**Figure 11:**
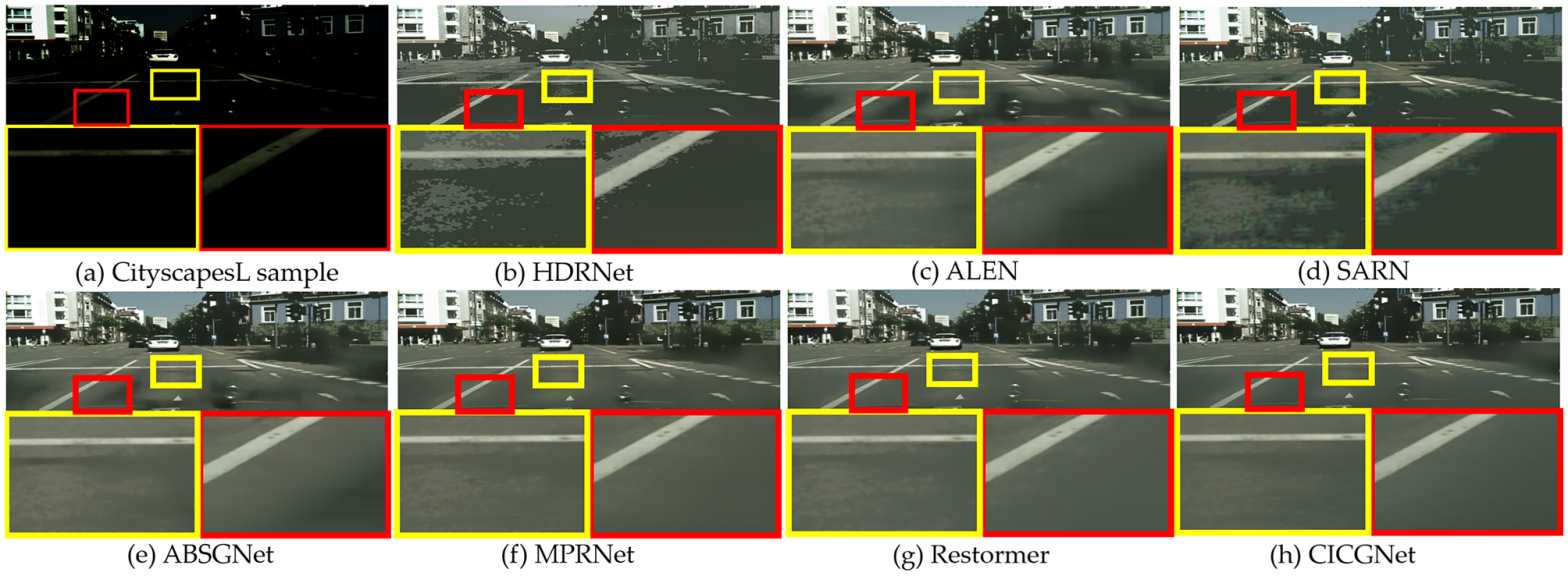
Visual results of low-light enhancement on CityscapesL.

**Figure 12:**
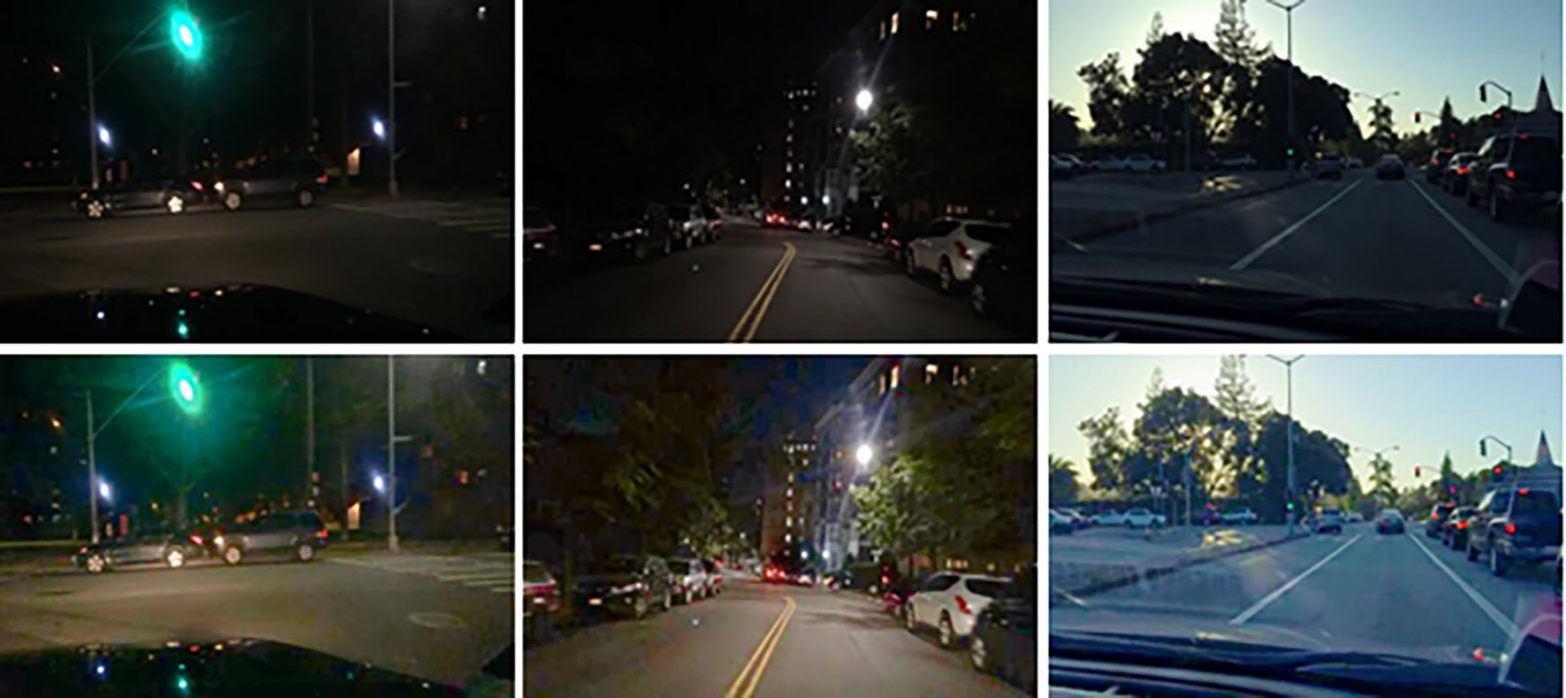
Enhanced visual effects of real night in BDD10K.

In this section, we show the visual enhancement effects of five test sample from Figs. 7, 8, 9, 10 and 11. As shown in Fig. 12, we also present the enhancement effect of using our proposed CICGNet on real night scenes in the BDD10K dataset.

**Figure 13:**
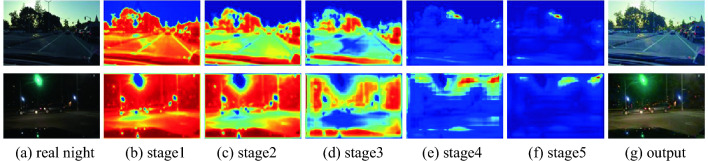
Stage-by-stage attention visualization.

As shown in Fig. 13, we give two sets of attention visualization results of real low-light samples in BDD10K, blue and red represent smaller and larger attention, respectively. We regard illumination component as attention along the width and depth of our proposed truss topology architecture. From stage1 to stage5, the early stage pays more attention to the global illumination map, the subsequent stages gradually tend to focus on the local illumination distribution.

### Ablation study

**Figure 14:**
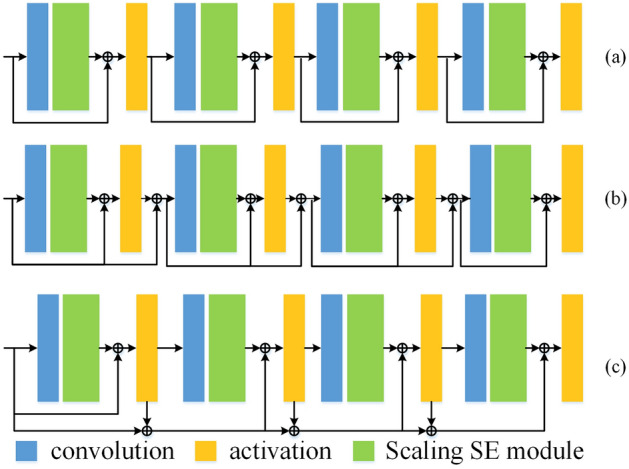
Different residual connection architectures.

We perform two sets of ablation study, firstly, we compare the performance of our proposed cascaded multi-residual architecture with two other residual connection ways in Table 7. These two compared residual architectures are shown in Fig. 14a and b, our proposed cascaded multi-residual architecture using pre- and post-activation features is shown in Fig. 14c. Secondly, we adjust the number of the feature decomposition and reconstruction. We perform the set of ablation study on CityscapesL, Table 8 shows the five indexes of image restoration quality, model size and inference time. We also present the effect of different scaling factors on model performance on the LOL in Table9. The scaling factors in the comparison experiments are 1, 4 (CICGNet), 8, and 16 respectively.

**Table 7:** The quantitative evaluation of different residual connection architecture.

Dataset	Architecture	PSNR↑	SSIM↑	LPIPS↓	deltaE↓	UQI↑
LOL	Fig. 14a	21.586	0.831	0.075	10.440	0.907
	Fig. 14b	21.344	0.829	0.078	10.671	0.908
	Fig. 14**c**	**22.420**	**0.894**	**0.073**	**8.940**	**0.921**
VELOL	Fig. 14a	20.914	0.828	0.106	11.629	0.924
	Fig. 14b	21.400	0.848	**0.068**	11.896	0.928
	Fig. 14**c**	**21.778**	**0.903**	0.070	**11.067**	**0.934**

Bold indicates the optimal value.

**Table 8:** Comparison of different number of decomposition and reconstruction.

Number of decomposition and reconstruction	Model↓/kb	CPU inference time↓/second	PSNR↑	SSIM↑	LPIPS↓	deltaE↓	UQI↑
1	**856**	**0.304**	23.761	0.886	0.177	12.100	0.916
3	2579	0.941	25.123	0.898	0.165	10.260	0.931
5	4333	1.622	25.383	0.901	0.162	9.816	0.936
7	6119	2.432	**26.017**	**0.905**	**0.159**	**9.111**	**0.942**

Bold indicates the optimal value.

**Table 9:** Comparison of different channel scaling factor on LOL.

Channel scaling factor\index	PSNR↑	SSIM↑	LPIPS↓	deltaE↓	UQI↑
CICGNet	**22.420**	**0.894**	**0.073**	**8.940**	**0.921**
Ratio1	22.156	0.837	0.075	9.054	0.915
Ratio8	21.346	0.832	0.077	10.483	0.908
Ratio16	21.454	0.833	0.075	10.634	0.906

Bold indicates the optimal value.

### Generalization

**Figure 15:**
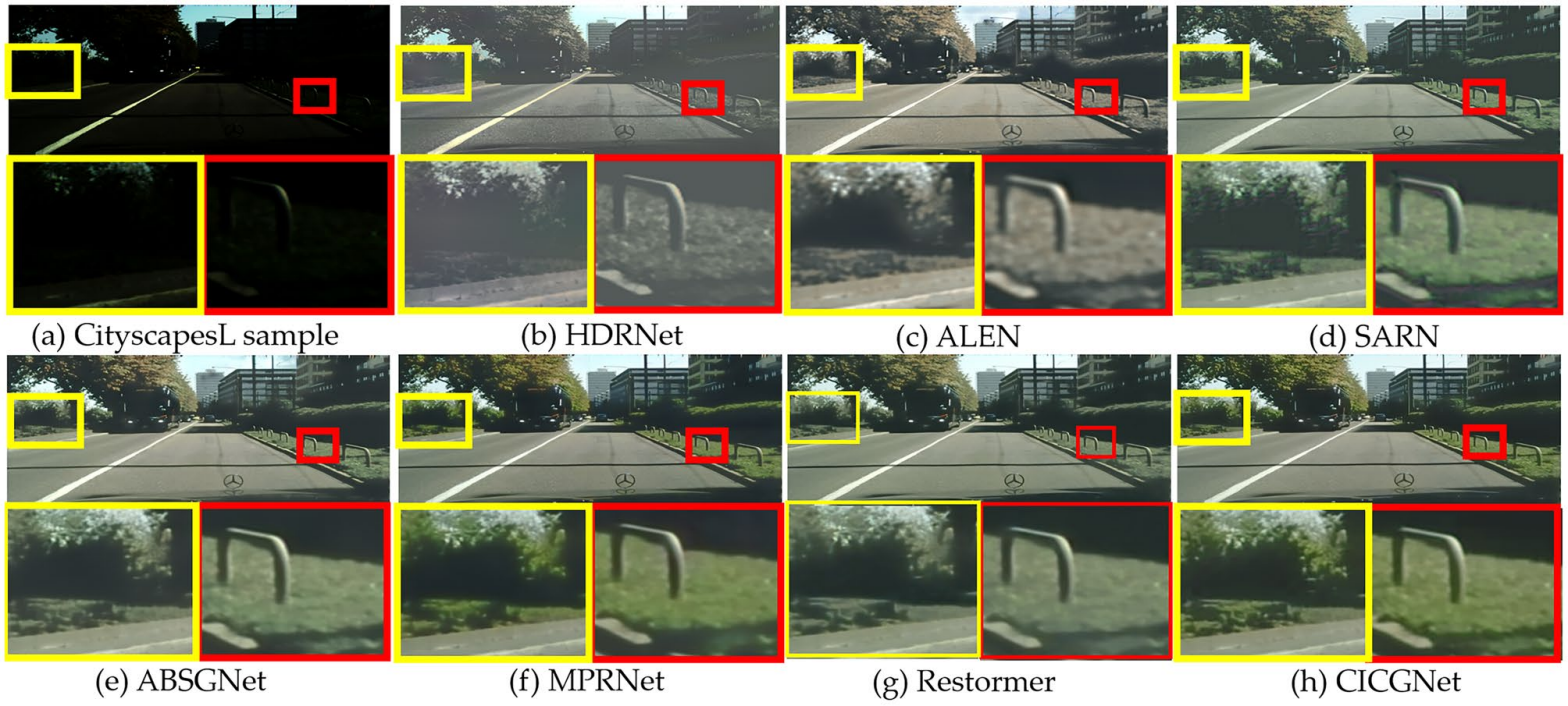
Visual evaluation of generalization on CityscapesL sample.

To evaluate the generalization of proposed CICGNet, we leverage the model trained on BDD10K_L^33^ to quantitatively and qualitatively evaluate the test set of CityscapesL. We give these results in Table 10 and Fig. 15.

**Table 10:** Quantitative evaluation of generalization on CityscapesL (1500 images).

Methods\index	PSNR↑	SSIM↑	LPIPS↓	deltaE↓	UQI↑
HDRNet	19.216	0.811	0.359	18.873	0.858
ALEN	21.585	0.851	0.264	14.661	0.897
SARN	21.416	0.841	*0.247*	15.121	0.892
ABSGNet	21.119	*0.857*	0.260	14.110	0.900
MPRNet	22.008	0.834	0.258	14.712	0.881
Restormer	*22.536*	0.856	0.249	**13.622**	*0.901*
CICGNet	**22.595**	**0.860**	**0.242**	*13.990*	**0.903**

Bold represents the optimal value, italics indicates the sub-optimal value.

### Application on semantic segmentation

**Figure 16:**
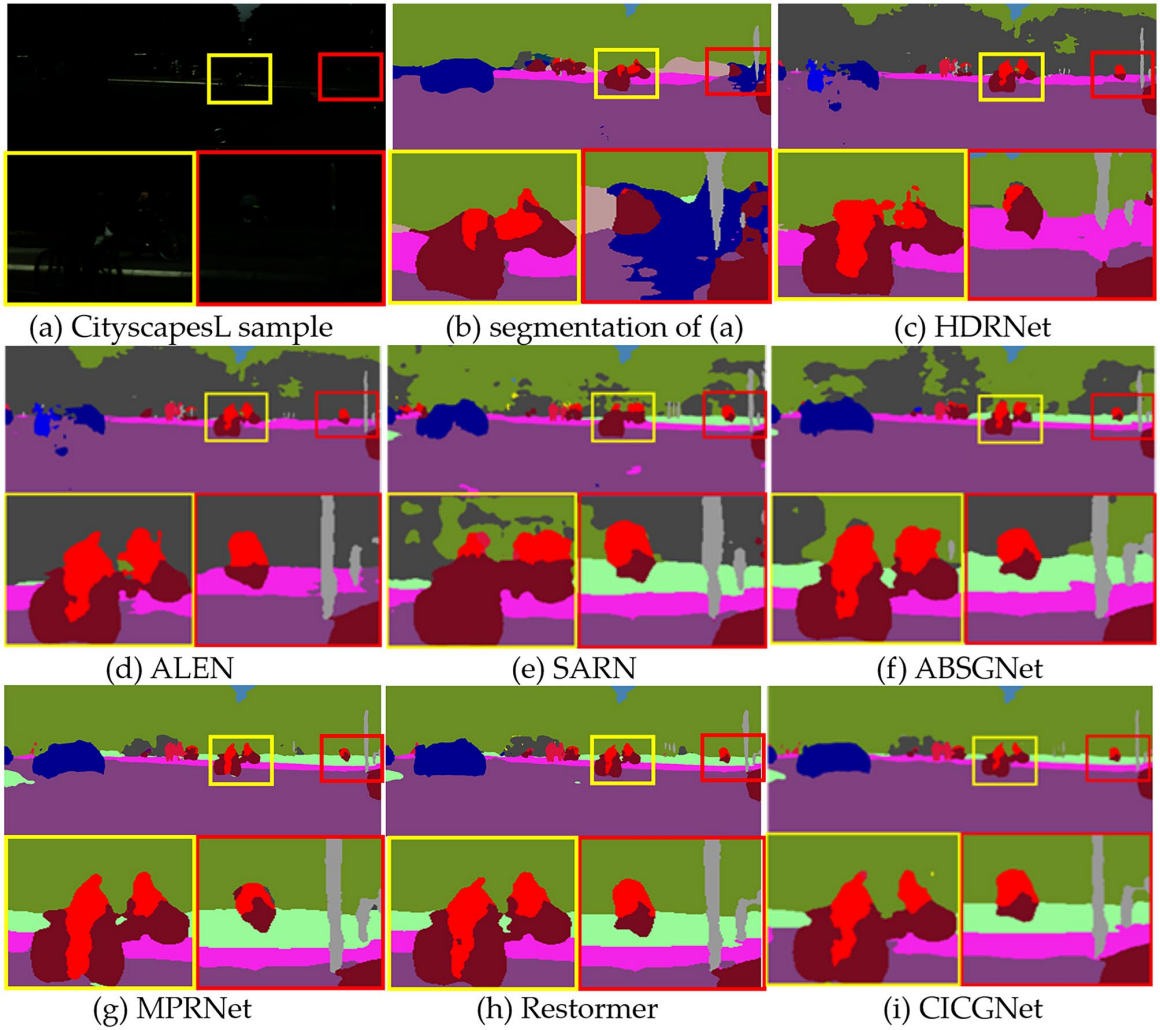
Segmentation visual results of CityscapesL processed by low-light enhancement algorithm.

To evaluate the effect of our proposed low-light enhancement algorithm on high-level vision task, we compare the effects of the above algorithms on semantic segmentation, we give their quantitative and qualitative results in Table 11 and Fig. 16. We leverage classic semantic segmentation DeepLabV3 + ^43^ to compare the above low-light enhancement algorithms. The evaluation result is on the default 19 categories. Table 11 shows mean pixel accuracy (mPA) and mean interaction over union (mIoU). As shown in Fig. 16, we show the segmentation visual results on the CityscapesL sample, including directly segmenting low-light samples, and using the above seven algorithms to enhance low-light images and then perform semantic segmentation.

**Table 11:** Comparison of semantic segmentation performance after processing with low-light enhancement algorithms (1500 images).

Methods\index	DeepLabv3 + _mobilenet		DeepLabv3 + _resnet101	
	mPA↑/(%)	mIoU↑/(%)	mPA↑/(%)	mIoU↑/(%)
Segmentation of low-light samples	29.10	23.00	34.40	26.85
HDRNet	21.55	16.76	27.23	19.62
SARN	33.37	26.90	*38.59*	29.84
ALEN	27.51	21.71	32.23	25.39
ABSGNet	34.85	25.69	37.03	28.43
MPRNet	34.66	25.31	37.46	28.77
Restormer	*34.91*	*27.35*	37.72	*30.31*
**CICGNet**	**36.68**	**30.51**	**38.76**	**32.79**

Bold represents the optimal value, italics indicates the sub-optimal value.

### Cascading optimization strategy

We report the results of semantic segmentation under different processing methods for low-light scenes in Table 12. We denote the segmentation model trained by CGNet^44^ on the original Cityscapes dataset (fine weather) as CityscapesSeg. Baseline represents segmentation of Cityscapes test sets, baseline0 indicates that low-light samples from CityscapesL are divided using CityscapesSeg, baseline1 uses the proposed low-light enhancement network CICGNet to enhance low-light samples and then uses CityscapesSeg for segmentation, baseline2 leverages the low-light samples to fine-tune the CityscapesSeg, the learning rate is 5 × 10^–5^, baseline3 represents cascade training low-light enhancement network and semantic segmentation network to form a unified cascade architecture.

**Table 12:** Comparison of semantic segmentation of different schemes for degraded samples.

Scheme	mPA↑/(%)	mIoU↑/(%)
Baseline	**65.64**	**56.06**
Baseline0	9.90	5.38
Baseline1	30.01	21.53
Baseline2	37.80	30.71
Baseline3	*38.16*	*31.05*

Bold represents the optimal value, italics indicates the sub-optimal value.

## Conclusion

Proposed low-light image enhancement is based on Retinex, it focuses on illumination component and content component along with depth and width directions of truss topology. We develop feature reuse concept to preserve content component and enhance illumination component in different truss branch. Comprehensive experiments show better performance in quantitative indexes and visual effects, compared with advanced attention-based low-light enhancement algorithms and state-of-the-art image restoration algorithms. We also perform several ablation studies, generalization experiment, and experiment on low-light enhancement algorithm applied to semantic segmentation.

## Data Availability

All data generated or analysed during this study are included in this published article.
